# Deoxynivalenol induces m^6^A-mediated upregulation of p21 and growth arrest of mouse hippocampal neuron cells in vitro

**DOI:** 10.1007/s10565-024-09872-7

**Published:** 2024-06-04

**Authors:** Peirong Xu, Yulan Zhao, Yue Feng, Mindie Zhao, Ruqian Zhao

**Affiliations:** 1 MOE Joint International Research Laboratory of Animal Health & Food Safety, Nanjing Agricultural University, Nanjing, Jiangsu People’s Republic of China; 2 Key Laboratory of Animal Physiology & Biochemistry, College of Veterinary Medicine, Nanjing Agricultural University, Nanjing, Jiangsu People’s Republic of China

**Keywords:** Deoxynivalenol, Proliferation, m^6^A, Ubiquitination, p21, Hippocampal neurons

## Abstract

Hippocampal neurons maintain the ability of proliferation throughout life to support neurogenesis. Deoxynivalenol (DON) is a mycotoxin that exhibits brain toxicity, yet whether and how DON affects hippocampal neurogenesis remains unknown. Here, we use mouse hippocampal neuron cells (HT-22) as a model to illustrate the effects of DON on neuron proliferation and to explore underlying mechanisms. DON exposure significantly inhibits the proliferation of HT-22 cells, which is associated with an up-regulation of cell cycle inhibitor p21 at both mRNA and protein levels. Global and site-specific m^6^A methylation levels on the 3’UTR of p21 mRNA are significantly increased in response to DON treatment, whereas inhibition of m^6^A hypermethylation significantly alleviates DON-induced cell cycle arrest. Further mechanistic studies indicate that the m^6^A readers YTHDF1 and IGF2BP1 are responsible for m^6^A-mediated increase in p21 mRNA stability. Meanwhile, 3’UTR of E3 ubiquitin ligase TRIM21 mRNA is also m^6^A hypermethylated, and another m^6^A reader YTHDF2 binds to the m^6^A sites, leading to decreased TRIM21 mRNA stability. Consequently, TRIM21 suppression impairs ubiquitin-mediated p21 protein degradation. Taken together, m^6^A-mediated upregulation of p21, at both post-transcriptional and post-translational levels, contributes to DON-induced inhibition of hippocampal neuron proliferation. These results may provide new insights for epigenetic therapy of neurodegenerative diseases.

**Graphical abstract:**

DON inhibits the proliferation of HT-22 cells.

RNA m^6^A hypermethylation on the transcript of p21 enhances the mRNA stability in a YTHDF1- and IGF2BP1-dependent manner, which leads to the upregulation of p21.

RNA m^6^A hypermethylation on the transcript of TRIM21 decreases the mRNA stability in a YTHDF2-dependent manner, which contributes to prevent p21 ubiquitin-mediated degradation.

High expression of p21 contributes to inhibit cell proliferation.

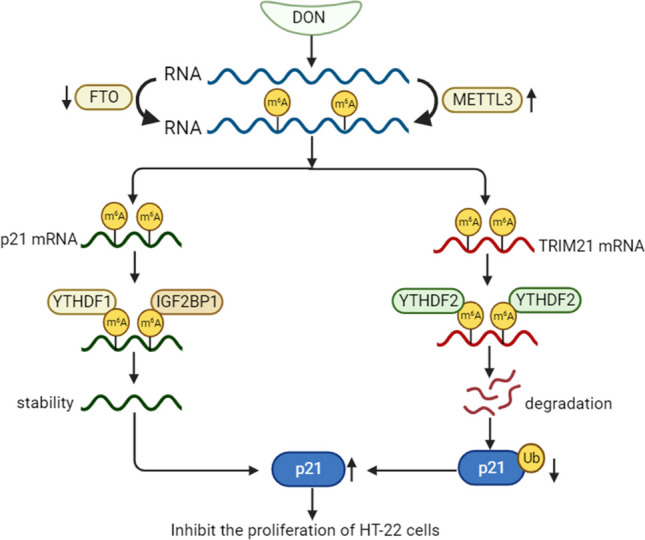

**Supplementary Information:**

The online version contains supplementary material available at 10.1007/s10565-024-09872-7.

## Introduction

Hippocampus is especially important for memory (Scoville and Milner 1957) and learning (Vila-Ballo et al. 2017), as well as cognition and emotions (Singhal et al. 2019). Hippocampus undergoes neurogenesis throughout the life (Eriksson et al. 1998). Disruption of hippocampal neurogenesis leads to various neurodegenerative diseases such as Alzheimer's disease (Donovan et al. 2006), depression (Malberg et al. 2000), and Parkinson's disease (Camicioli et al. 2003). Hippocampal neurogenesis involves coordinated processes including proliferation, differentiation and migration of neural precursor cells (Llorens-Martin et al. 2015). Cell proliferation is tightly controlled by cell cycle progression, which is mediated by cyclin-dependent kinases (CDKs) and their regulatory cyclins. Cyclin/CDK complexes are regulated by both stimulatory and inhibitory pathways (Schafer 1998). p21 is a potent inhibitor of cyclin-CDK complexes, halting cell cycle progression in G1 phase and leading to inhibition of cell proliferation (Harper et al. 1993). p21 plays a key role in inhibiting neuronal cell proliferation. For instance, upregulation of p21 leads to inhibition of mouse neural stem cell (NSCs) proliferation (Maeda et al. 2023), whereas in p21-null mice, neural precursor cell proliferation in the hippocampus was increased after cerebral ischemia (Qiu et al. 2004). p21 is subjected to transcriptional, post-transcriptional, and post-translational regulation. For instance, the tumor suppressor protein p53 directly transactivates p21 to cause cell cycle arrest (el-Deiry et al. 1993, Waldman et al. 1995). miR-106b binds to 3'UTR of p21 mRNA to degrade p21 mRNA and promote cell cycle progression (Ivanovska et al. 2008). Moreover, preventing p21 protein ubiquitination inhibits the proliferation of hepatocellular carcinoma cells (Huang et al. 2022).

m^6^A is the most prevalent modification in eukaryotic mRNAs (Zhao et al. 2017), which is dynamically and reversibly regulated by methyltransferases (“writers”) including METTL3 and METTL14, as well as demethylases (“erasers”) including ALKBH5 and FTO (Fu et al. 2014). Specific m^6^A binding proteins (“readers”), such as YTHDF1, YTHDF2 and IGF2BP1, can recognize m^6^A marks and regulate the fate of the mRNAs in a context-specific manner during cellular stress responses (Shi et al. 2019). The function of RNA m^6^A methylation in hippocampal neurogenesis has been inconsistent. For instance, FTO deficiency reduces the neuronal proliferation and differentiation of adult hippocampal neural stem cells in mice, resulting in learning and memory impairment (Li et al. 2017). Instead, interrupting m^6^A-mediated regulation by knockdown of METTL3 also causes aberrant cell cycle events in hippocampal pyramidal neurons, leading to significant memory deficits (Zhao et al. 2021).

Previous studies indicate that m^6^A is also involved in mycotoxin-induced cellular responses. For instance, Aflatoxin B1 exposure causes global m^6^A hypomethylation in bovine mammary epithelial cells in association with cell cycle arrest and apoptosis activation (Wu et al. 2021). Also, AFB1-induced oxidative stress in mouse liver is accompanied by FTO down-regulation and global m^6^A hypermethylation (Wu et al. 2020). Deoxynivalenol (DON) is a mycotoxin in grains and poses a considerable risk to human and animal health when present in food and feed (Kamle et al. 2022). DON can cause oxidative stress, neurotransmitter disruption, and cell apoptosis in the brain (Zhang et al. 2020). For instance, DON triggers lipid peroxidation in the hippocampus of piglets, which is associated with dampened neurotransmitter levels (Wang et al. 2020a). DON also induces apoptosis and autophagy in piglet hippocampal neuronal cells (Wang et al. 2020b). Nevertheless, it remains unexplored whether RNA m^6^A modification is involved in DON-induced hippocampal neuron toxicity.

In this study, we provide evidence that DON induces G0/G1 arrest in HT-22 cells through upregulation of p21, which involves m^6^A-mediated post-transcriptional and post-translational regulation exerted by different “readers” on different target transcripts. These findings demonstrate the crucial role of RNA m^6^A methylation in DON-induced inhibition of hippocampal neuron proliferation and provide new insights into developing targeted therapies to treat neurodegenerative diseases.

## Materials and methods

### Cell culture and treatment

HT-22 cells (undifferentiated) were obtained from Shanghai HuiYing Biological Technology Co. Ltd (China) and cultured in DMEM medium (319–005, Wisent, Canada) supplemented with 10% FBS (FS301-02, TransGen, China) and 1% penicillin/streptomycin (BC-CE-007, Biochannel, China) at 37 °C in 5% CO_2_. HT-22 cells were cultured to 80% confluency and then treated with 1 or 2 μM DON (MSS1011, Pribolab, Singapore) for 12 h. Cell viability was detected by CCK-8 (FC101, TransGen, China).

### EdU click chemistry assay and fluorescence imaging

EdU (5-ethynyl-20-deoxyuridine) cell proliferation kit (G1601, Servicebio, China) was used to test cell proliferation. Generally, cells were treated with DON for 12 h before adding 100 μL of 10 μM EdU. Cells were maintained in culture for another 1.5 h, and then were fixed with 4% paraformaldehyde solution for 15 min. The cells were then permeabilized with 0.25% Triton-X-100 in PBS for 15 min. Then cells were incubated in the dark for 30 min with a reaction cocktail containing the compounds necessary for the binding Alexa Fluor® 488 azide with EdU. Finally, the nuclei were stained with Hoechst 33,342 for 10 min. Fluorescence microscope was used to collect images for calculating the cell proliferation rate.

### Cell apoptosis and cycle analysis

For the apoptosis assay, cells were collected with EDTA-free trypsin solution (BL527A, Biosharp, China), and suspended in binding buffer containing Annexin V-iFluor 488 and PI (G1513, Servicebio, China). Cell fluorescence was detected by flow cytometry (FACS Verse™, BD Biosciences, USA). For the analysis of the cell cycle, cells were harvested with trypsin solution (BL512B, Biosharp, China), and then fixed in 75% ethanol overnight at -20 °C. Cells were then stained with propidium iodide (PI)/RNase mixture in the dark for 40 min at 37 °C (G1021, Servicebio, China). Flow cytometry (FACS Verse™, BD Biosciences, USA) was used to analyze the results.

### Luciferase activity assay

Plasmids carrying wild-type p21-3’UTR (p21-3’UTR-WT) and m^6^A site mutated p21-3’UTR (p21-3’UTR-Mut) were constructed using pmiGLO vector. The Dual-Luciferase Reporter Assay System (Tsingke Biotech, China) was used for the luciferase reporter assay. Then cells were transfected with respective plasmids for 12 h and treated with or without DON for another 12 h. The activity of firefly luciferase was normalized to the activity of Renilla luciferase to reflect the transfection efficiency.

### RNA extraction and qRT–PCR

Total RNA extraction from HT-22 cells using Total RNA Extraction Reagent (R401-01, Vazyme Biotech, China), and the cDNA was synthesized using the Reverse Transcription Kit (AU341, TransGen Biotech, China). Gene expression was assessed using a QuantStudio™ 6 Flex real-time PCR system (Thermo Scientific, USA). Supplementary Table 1 provides information about the primers used.

### Western blot analysis

Total proteins extraction from HT-22 cells using RIPA lysis buffer (BD0032, Bioworld, China) with protease inhibitor cocktail (b14001, Selleckchem, USA). The BCA Protein Assay Kit (DQ111, TransGen Biotech, Beijing) was used to measure protein concentration. Protein samples were loaded onto a sodium dodecyl sulfate polyacrylamide gel and electrophoresed before transfer to a nitrocellulose membrane. Supplementary Table 2 contains information on the antibodies that were used. Bands were visualized by chemiluminescent reaction (BL520B, Biosharp, China). Image J software was used to analyze the results.

### RNA m^6^A dot blot assay

The isolated RNA (500 ng) was incubated at 95 °C for 10 min and added on Hybond-N^+^ membrane. After 5 min of UV cross-linking, the membrane was stained with methylene blue solution for 5 min, washed with TBST buffer for 5 min, and images were taken as loading controls. The membrane was incubated with anti-m^6^A antibody (68,055–1-Ig, Proteintech, diluted 1:2000) overnight at 4 °C after blocking in 5% nonfat dry milk. Then, the membrane was incubated with goat anti-mouse IgG (BL001A, Biosharp, China, diluted 1:200,000) at room temperature for 2 h. Finally, bands were observed by chemiluminescence reaction (BL520B, Biosharp, China). Image J software was used to analyze the results.

### Ubiquitination analysis

Cells were treated with 20 μM proteasome inhibitor MG132 (HY-13259, MCE, USA) for 6 h before being harvested using RIPA lysis buffer (BD0032, Bioworld, USA) containing protease inhibitor cocktail (b14001, Selleckchem, USA). The protein (200 μg) was incubated with anti-p21 antibody (3 μg; 10,355–1-AP, Proteintech, USA) and protein A/G agarose (BD0048, Bioworld, China) overnight at 4 °C. Normal IgG (BD0051, Bioworld, USA) was included as a negative control. Then, PBS was used to wash the beads, and samples were collected. The collected samples was further separated on an SDS-PAGE gel. Anti-ubiquitin antibody (Ab134953, Abcam, USA) was then used for immunoblotting.

### SELECT for Site-specific detection of m^6^A

Sequences of p21 mRNA were subjected to specific m^6^A site prediction using SRAMP (http://www.cuilab.cn/sramp). Four m^6^A sites predicted with very high, high and modest confidence were verified using the SELECT method as previously described (Xiao et al. 2018). Supplementary Table 3 provides information on the specific primers used for SELECT.

### Cell transfection

The sequences of the siRNAs are as follows: siMETTL3: 5'-CCUCAGUGGAUCUGUUGUGAU-3' (Tsingke Biotech, China), siYTHDF1: 5'-GCCCACAGCUAUAACCCUAAA-3' (Tsingke Biotech, China), siYTHDF2: 5'-CCAUGCCCUAUCUAACUUCUU-3' (Tsingke Biotech, China) and siIGF2BP1: 5'-GGCCAGUUCUUGGUCAAAU-3' (GenePharma, China). Cell transfection was performed by using the JetPRIME® transfection reagent (101,000,046, Polyplus, France). Scrambled siRNA was used as the negative control (siNC).

### RNA and protein stability analysis

For RNA decay analysis, cells were cultured with 2 μM DON for 12 h and then treated with 5 µg/mL actinomycin D (HY-17559, MCE, USA) for 0, 4 and 8 h to inhibit the de novo transcription. Total RNA extraction from cells using Total RNA Extraction Reagent. After reverse transcription, target mRNA levels were detected by qRT-PCR (relative to 0 h). For protein decay analysis, cells were treated with 10 μM protein synthesis inhibitor cycloheximide (40325ES03, Yeasen Biotechnology, China) for various periods of time. Total protein was then extracted and analyzed by Western blot.

### Statistical analysis

Graph Prism 8 software (GraphPad Software Inc., San Diego, CA, USA) was used for the statistical analysis. In addition, the experiments were conducted at least three times. Differences between groups were analyzed using Student’s t-test (two-group comparison) or one-way ANOVA (more than two groups) followed by Bonferroni multiple comparison test. All data are expressed as mean ± SD, and *P* < 0.05 is considered a statistically significant difference. *P < 0.05, **P < 0.01, “ns” indicates no statistical difference.

## Results

### DON-induced inhibition of HT-22 cell proliferation is associated with upregulation of p21

**Fig. 1:**
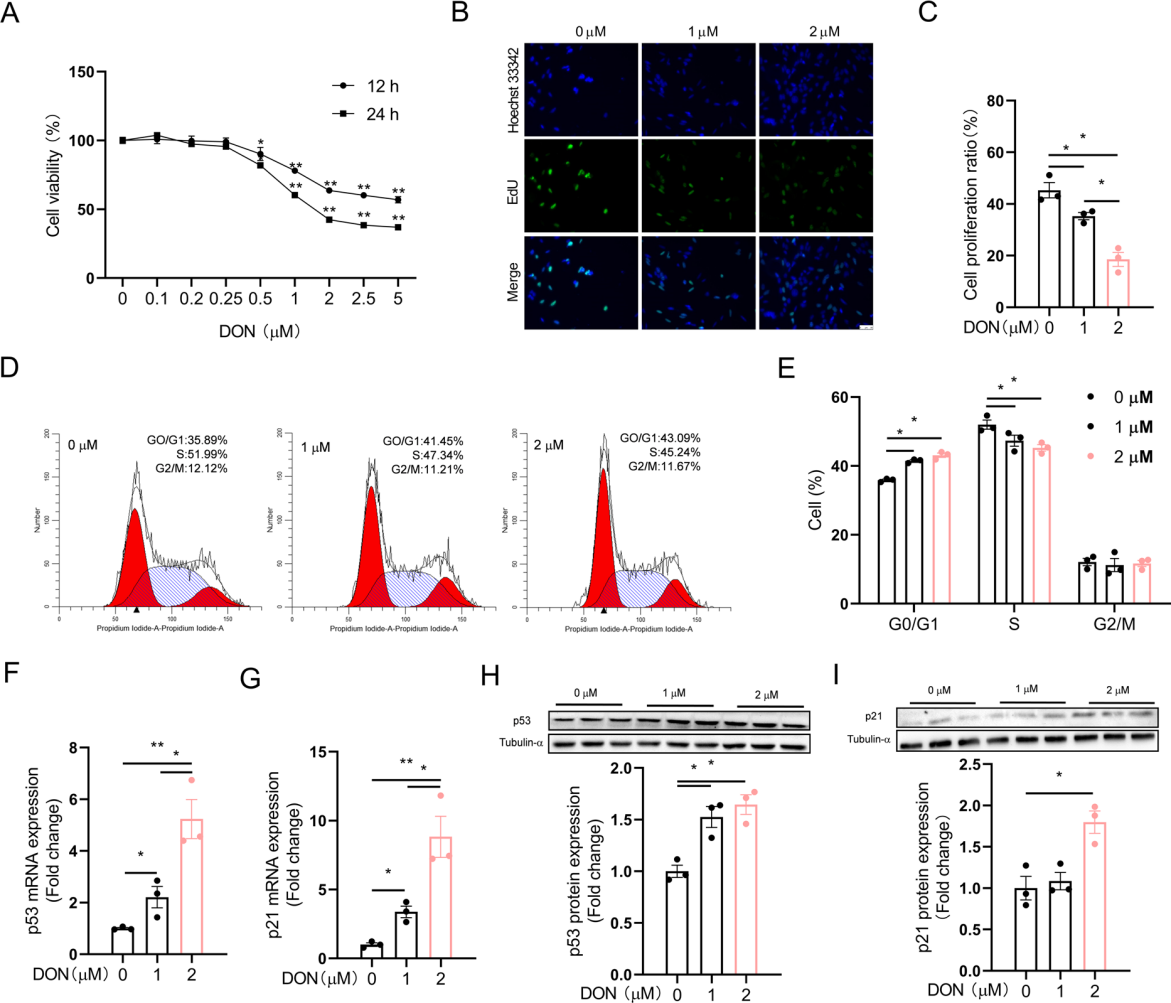
DON inhibits the proliferation of HT-22 cells. HT-22 cells were treated with DON (1 or 2 μM) for 12 h. **A** Cell viability (*n* = 5). **B**, **C** EdU incorporation assays (*n* = 3). **D**, **E** Cell cycle assays (*n* = 3). **F**, **G** p53 and p21 mRNA expression (*n* = 3). **H**, **I** p53 and p21 protein expression (*n* = 3). The differences between groups were analyzed using one-way ANOVA with Bonferroni’s correction. Values are means ± SD, *P < 0.05, **P < 0.01

Cell viability was significantly decreased in a dose- and time-dependent manner after DON treatment (Fig. 1A), which is confirmed by morphological observation of fewer cell numbers in DON-exposed groups (Fig. S1A). Further fluorescence microscopy (Fig. S1B, C) and flow cytometry (Fig. S1D, E) assays indicate no significant differences in apoptosis rates. Whereas EdU imaging indicates significantly decreased cell proliferation ratios after DON treatment (Fig. 1B, C). Cell cycle analysis reveals an arrest at G0/G1 phase (Fig. 1D, E). Concurrently, the key cell proliferation regulators, p53 and p21, were both upregulated significantly at mRNA and protein levels (Fig. 1F-I). These findings indicate that DON inhibits HT-22 cell proliferation.

### DON-induced inhibition of HT-22 cell proliferation requires m^6^A hypermethylation

**Fig. 2:**
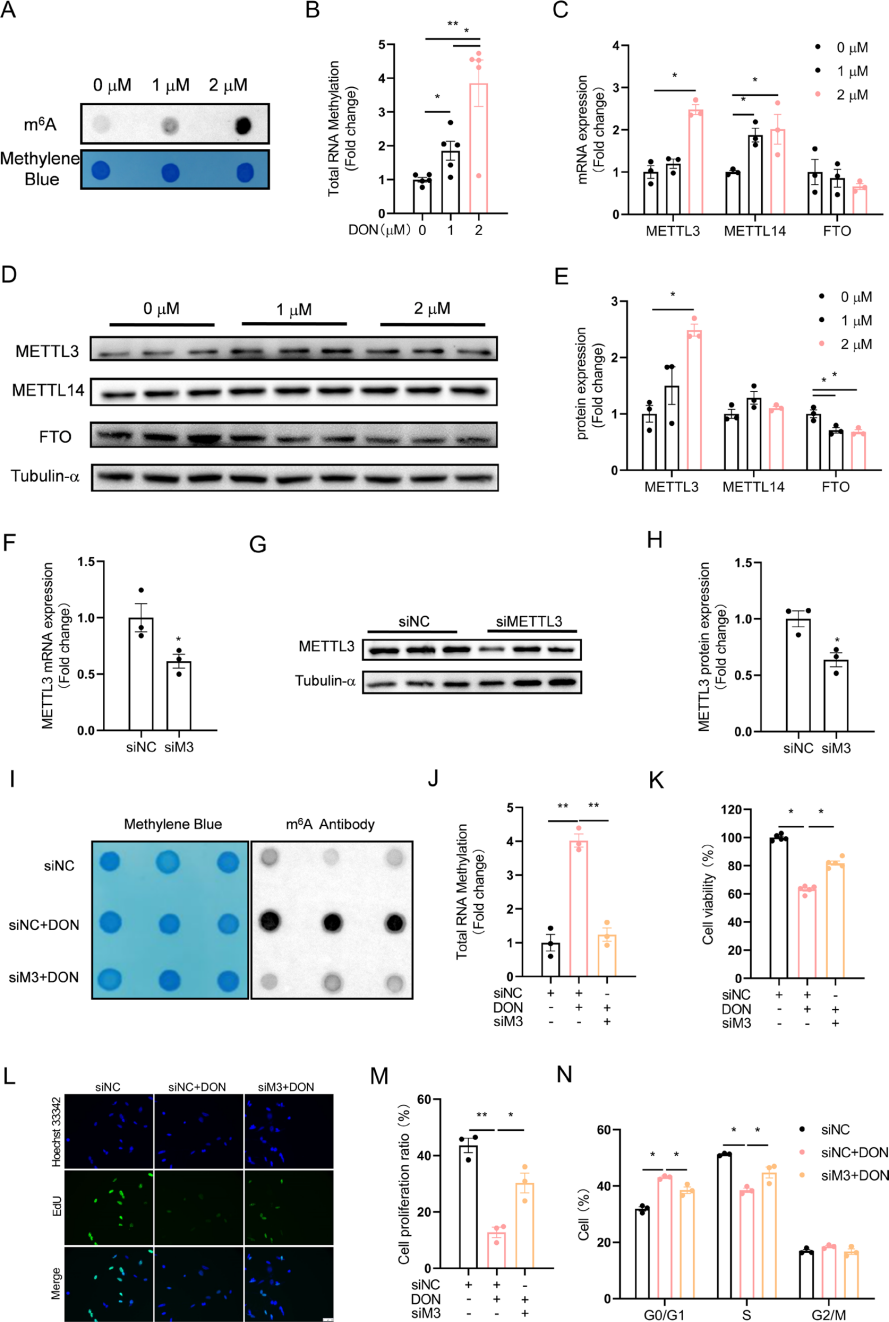
DON-induced inhibition of HT-22 cell proliferation requires m^6^A hypermethylation. HT-22 cells were treated with DON (1 or 2 μM) for 12 h. **A**, **B** Total RNA m^6^A modification (*n* = 5). The differences between groups were analyzed using one-way ANOVA with Bonferroni’s correction. **C**-**E** METTL3, 14 and FTO mRNA and protein expression (*n* = 3). The differences between groups were analyzed using one-way ANOVA with Bonferroni’s correction. **F–H** Inhibition of METTL3 mRNA and protein expression by METTL3 siRNA transfection (*n* = 3). The differences between groups were analyzed using student’s t test. **I**, **J** Inhibition of total RNA m^6^A modification by METTL3 siRNA transfection (*n* = 3). The differences between groups were analyzed using one-way ANOVA with Bonferroni’s correction.** K** Effect of METTL3 siRNA on cell viability (*n* = 5). The differences between groups were analyzed using one-way ANOVA with Bonferroni’s correction. **L**,** M** Effect of METTL3 siRNA on cell proliferation ratio (*n* = 3). The differences between groups were analyzed using one-way ANOVA with Bonferroni’s correction. **N** Effect of METTL3 siRNA on cell cycle distribution (*n* = 3). The differences between groups were analyzed using one-way ANOVA with Bonferroni’s correction. Values are means ± SD, *P < 0.05, **P < 0.01

Global RNA m^6^A levels of total RNA extracted from DON-treated cells were significantly increased as shown in dot blot (Fig. 2A, B). Accordingly, METTL3 and METTL14 were significantly increased in DON-exposed cells at mRNA and/or protein levels, while the demethylase FTO was significantly decreased at protein level (Fig. 2C-E). METTL3 knockdown (Fig. 2F-H) significantly alleviated DON-induced m^6^A hypermethylation (Fig. 2I, J), which was associated with significantly mitigating the abnormal cell morphology (Fig. S2A), impaired cell viability (Fig. 2K), inhibited proliferation (Fig. 2L, M) and cell cycle arrest (Fig. 2N and S2B). These results indicate that m^6^A modification is involved in DON-induced inhibition of HT-22 cell proliferation.

### Specific m^6^A site in 3’UTR of p21 mRNA contributes to DON-induced p21 upregulation

**Fig. 3:**
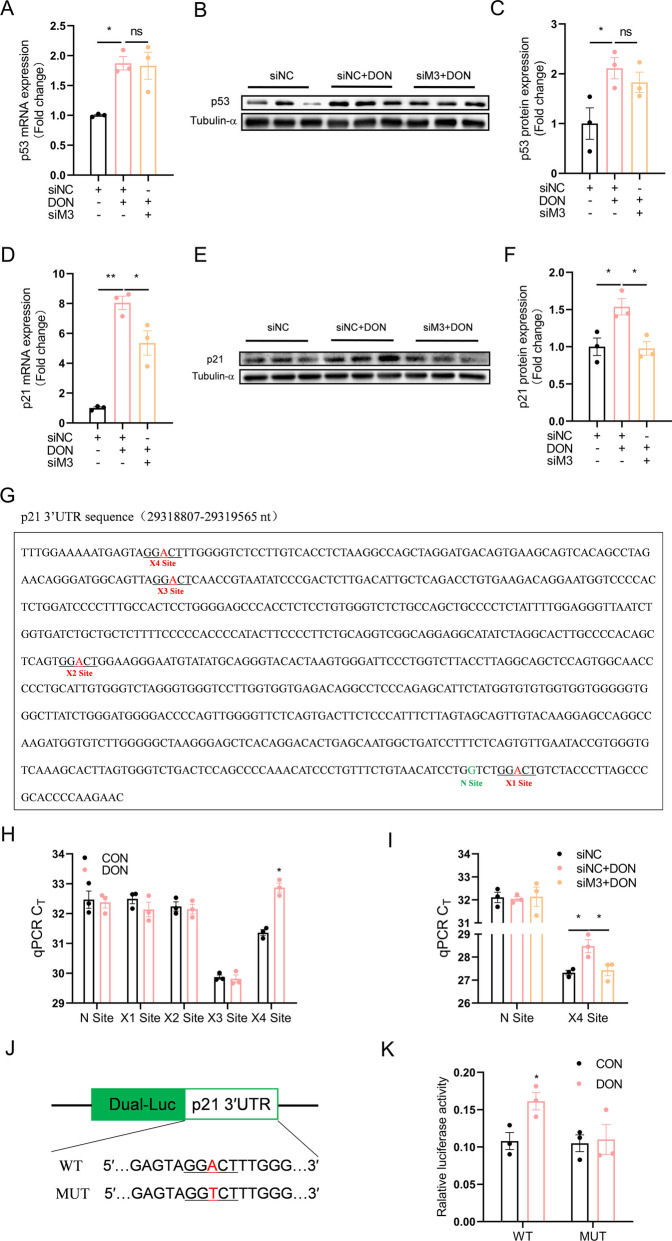
Specific m^6^A site in 3’UTR of p21 mRNA contributes to DON-induced p21 upregulation. HT-22 cells were treated with DON (2 μM) for 12 h. **A**-**C** Effect of METTL3 siRNA on p53 mRNA and protein expression (*n* = 3). The differences between groups were analyzed using one-way ANOVA with Bonferroni’s correction. **D**-**F** Effect of METTL3 siRNA on p21 mRNA and protein expression (*n* = 3). The differences between groups were analyzed using one-way ANOVA with Bonferroni’s correction. **G** m^6^A site was predicted in SRAMP for p21 3’UTR 29318807–29319565 nt sequence. RRACU-compliant motif was named motif 1–4. A very high, high, and moderate confidence site “A” was obtained and this “A” was named X site. The non-modification A was named N site. **H** Validation of m^6^A modification in p21 3’UTR using SELECT (*n* = 3). X site was predicted by SRAMP and N site (non-modification site) was negative control (*n* = 3). The differences between groups were analyzed using student’s t test. **I** Effect of METTL3 siRNA on m^6^A modification on X4 site of p21 3’UTR using SELECT (*n* = 3). The differences between groups were analyzed using one-way ANOVA with Bonferroni’s correction. **J** Schematic representation of mutated (GGACT to GGTCT, red dots) 3’UTR of pmiGLO-Basic vector to investigate the m^6^A roles on p21 expression. **K** Dual-luciferase activity of pmiGLO-Basic-3’UTR WT or pmiGLO-Basic-3’UTR MUT4 reporter were transfected into CON or DON treated HT-22 cells (*n* = 3). The differences between groups were analyzed using one-way ANOVA with Bonferroni’s correction. Values are means ± SD, *P < 0.05, **P < 0.01, “ns” indicates no statistical difference

p53 is an oncogenic protein that regulates apoptosis and cell cycle by transactivating p21 (Hernandez Borrero and El-Deiry 2021). However, knockdown of METTL3 had no effects on DON-induced p53 upregulation (Fig. 3A-C), while significantly attenuating DON-induced upregulation of p21 mRNA (Fig. 3D) and protein (Fig. 3E, F). Therefore, p21, not p53, is likely the target for m^6^A-mediated regulation. UC2288, a p21 inhibitor, significantly alleviated DON-induced abnormal cell morphology (Fig. S3A), reduced cell viability (Fig. S2B), inhibited proliferation (Fig. S3C, D) and cell cycle arrest (Fig. S3E, F). Specific m^6^A sites were predicted in 3’UTR of p21 mRNA using SRAMP (http://www.cuilab.cn/sramp) (Fig. 3G and Supplementary Table 4). Four m^6^A modification sites (X1-X4) with very high, high, and moderate confidence were verified with SELECT relative to the negative control site (N site). Only X4 site in p21 mRNA 3'UTR showed significantly increased level of m^6^A modification upon DON exposure (Fig. 3H), which was prevented by METTL3 knockdown (Fig. 3I). Luciferase reporter assay with plasmids (Fig. 3J) carrying wild-type (WT) and X4 site mutated (MUT) p21-3’UTR further confirmed the role of X4 site in DON-induced p21 up-regulation, as X4 site mutation abolished DON-induced increase in luciferase activity (Fig. 3K), suggesting that m^6^A modification on this specific site is indispensable for p21 up-regulation in DON-treated HT-22 cells.

### YTHDF1 and IGF2BP1 contributes to m^6^A-mediated increase in p21 mRNA stability

**Fig. 4:**
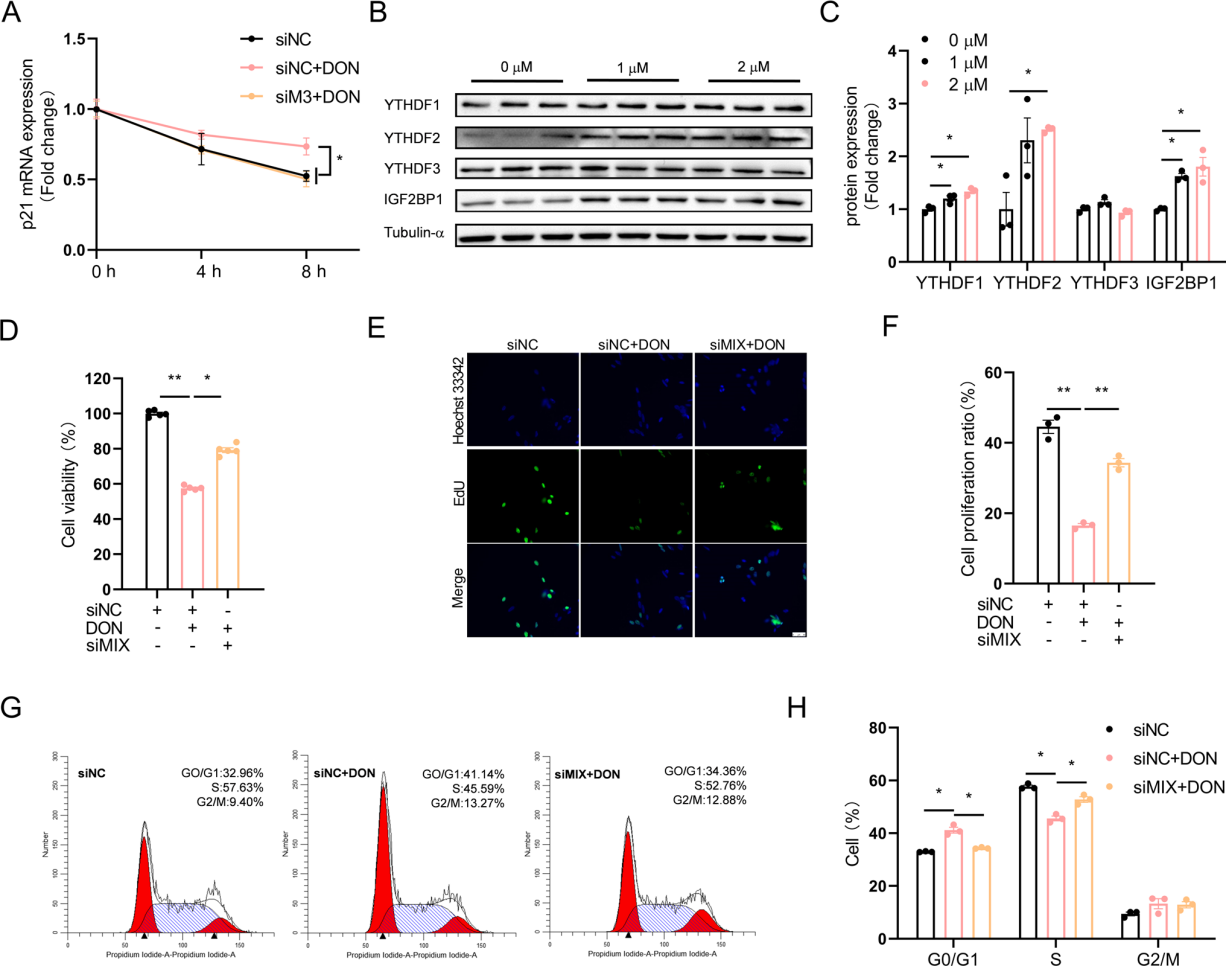
Simultaneous knockdown of YTHDF1, YTHDF2 and IGF2BP1 attenuates DON-induced proliferation inhibition. HT-22 cells were treated with DON (1 or 2 μM) for 12 h. **A** Effect of METTL3 siRNA on p21 mRNA stability (*n* = 3). **B**, **C** YTHDF1, 2, 3 and IGF2BP1 protein expression (*n* = 3). **D** Effect of MIX siRNA on cell viability (*n* = 5). siMIX: siYTHDF1 + siYTHDF2 + siIGF2BP1. **E**, **F** Effect of MIX siRNA on cell proliferation ratio (*n* = 3).** G**, **H** Effect of MIX siRNA on cell cycle distribution (*n* = 3). The differences between groups were analyzed using one-way ANOVA with Bonferroni’s correction. Values are means ± SD, *P < 0.05, **P < 0.01

**Fig. 5:**
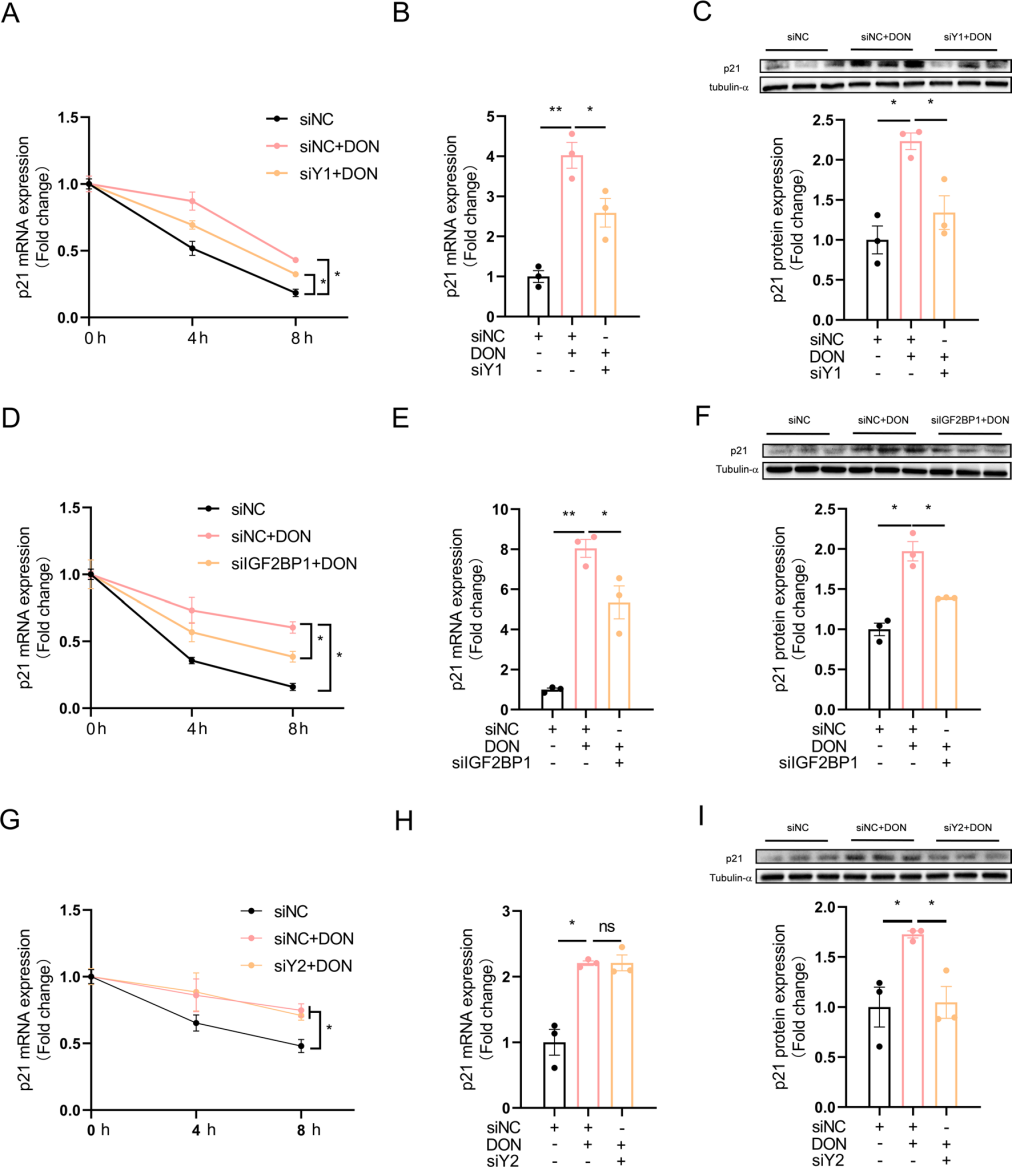
Effects of individual knockdown of YTHDF1, YTHDF2, or IGF2BP1 on p21 mRNA stability. HT-22 cells were treated with DON (2 μM) for 12 h. **A** Effect of YTHDF1 siRNA on p21 mRNA stability (*n* = 3). **B**, **C** Effect of YTHDF1 siRNA on p21 mRNA and protein expression (*n* = 3). **D** Effect of IGF2BP1 siRNA on p21 mRNA stability (*n* = 3). **E**, **F** Effect of IGF2BP1 siRNA on p21 mRNA and protein expression (*n* = 3). **G** Effect of YTHDF2 siRNA on p21 mRNA stability (*n* = 3). **H**, **I** Effect of YTHDF2 siRNA on p21 mRNA and protein expression (*n* = 3). The differences between groups were analyzed using one-way ANOVA with Bonferroni’s correction. Values are means ± SD, *P < 0.05, **P < 0.01, “ns” indicates no statistical difference

To investigate whether m^6^A modification increases mRNA stability as previously reported (Zhang et al. 2022a), we conducted the mRNA stability using transcriptional repressor actinomycin D. The stability of p21 mRNA was significantly increased in DON-treated HT22 cells, and METTL3 knockdown significantly prevented DON-induced increase in p21 mRNA stability (Fig. 4A). Three out of the 4 m^6^A “readers”, which includes YTHDF1, YTHDF2 and IGF2BP1, were significantly increased in DON-treated HT22 cells (Fig. 4B, C). Simultaneous knockdown of these 3 “readers” significantly alleviated DON-induced reduction in cell viability (Fig. 4D) and proliferation ratio (Fig. 4E, F) and rescued cell cycle arrest (Fig. 4G, H) in DON-treated HT22 cells. Knockdown of YTHDF1 (Fig. S4A, B), YTHDF2 (Fig. S4C, D) or IGF2BP1 (Fig. S4E, F) individually, partly yet significantly alleviated DON-induced cellular toxicity, as indicated in cell viability (Fig. S5A, F, K), proliferation ratio (Fig. S5B, C, G, H, L, M) and cell cycle arrest (Fig. S5D, E, I, J, N, O). Interestingly, individual knockdown of these 3 “readers” showed different effects on p21 expression. YTHDF1 or IGF2BP1 knockdown partly yet significantly rescued DON-induced increase in p21 mRNA stability (Fig. 5A, D), as well as p21 mRNA (Fig. 5B, E) and protein (Fig. 5C, F) levels. However, YTHDF2 knockdown did not affect p21 mRNA stability (Fig. 5G) or mRNA level (Fig. 5H), but significantly alleviated DON-induced increase of p21 protein content (Fig. 5I) in HT22 cells.

### YTHDF2 contributes to m^6^A-mediated increase in p21 protein stability via ubiquitination-dependent mechanism

**Fig. 6:**
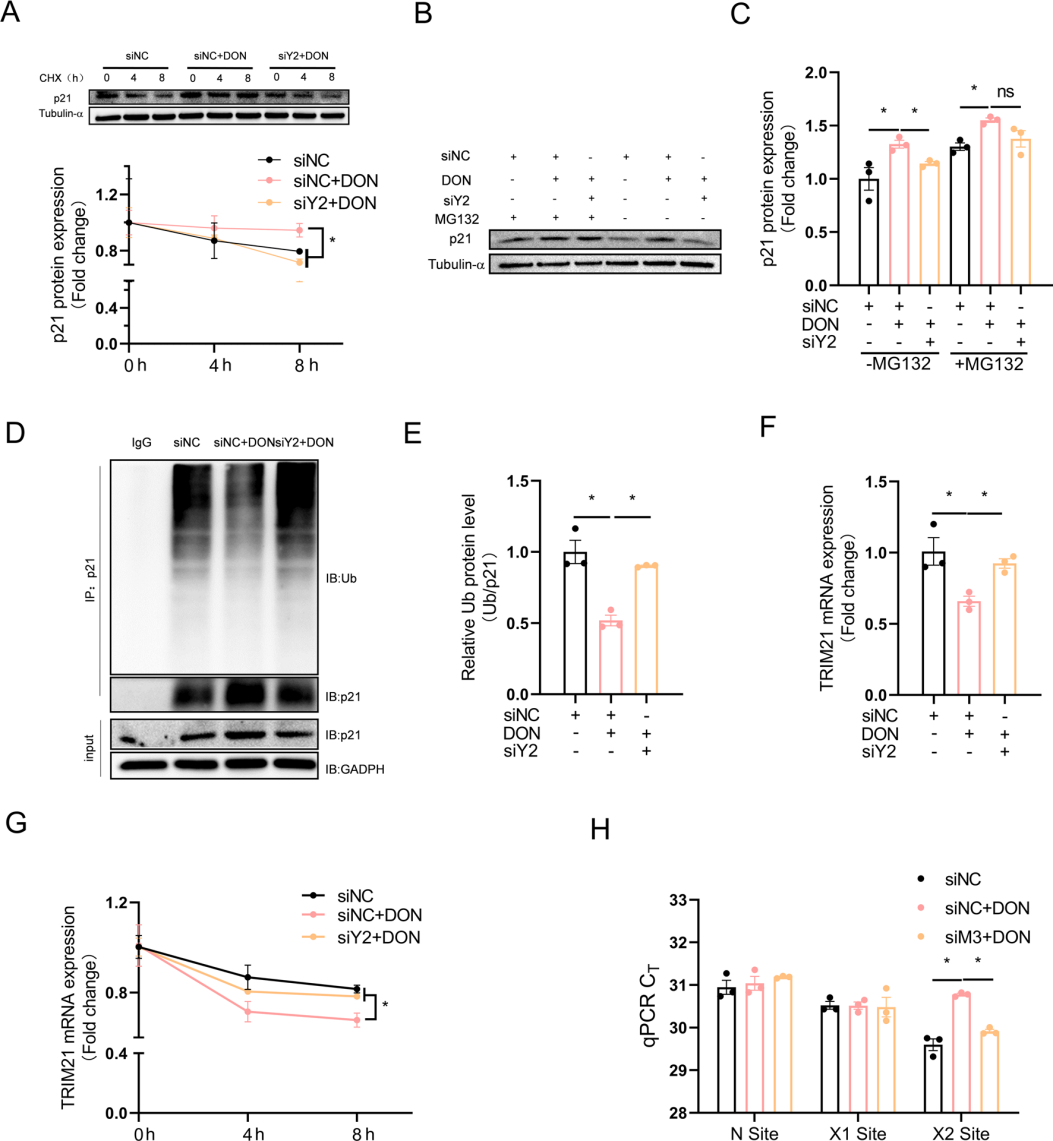
YTHDF2 contributes to m^6^A-mediated increase in p21 protein stability via ubiquitination-dependent mechanism. HT-22 cells were treated with DON (2 μM) for 12 h. **A** Effect of YTHDF2 siRNA on p21 protein stability (*n* = 3). **B**, **C** Cells transfected with YTHDF2 siRNA and treated with DON and then were treated with MG132 (20 μM) for 6 h before harvesting, and the expression of p21 was analyzed by Western blotting (*n* = 3). **D**, **E** Cells transfected with YTHDF2 siRNA and treated with DON and then were treated with MG132 (20 μM) for 6 h before harvesting, and the ubiquitination level of endogenous p21 was measured using an ubiquitination assay (*n* = 3). **F** Effect of YTHDF2 siRNA on TRIM21 mRNA expression (*n* = 3). **G** Effect of YTHDF2 siRNA on TRIM21 mRNA stability (*n* = 3). **H** Validation of m^6^A modification in TRIM21 using SELECT. X site was predicted by SRAMP and N site (non-modification site) was negative control (*n* = 3). The differences between groups were analyzed using one-way ANOVA with Bonferroni’s correction. Values are means ± SD, *P < 0.05, “ns” indicates no statistical difference

The p21 protein is unstable and can be rapidly degraded by ubiquitination (Geng et al. 2022). To determine whether DON-induced upregulation of p21 involves improved protein stability, We conducted the half-life of p21 protein using cycloheximide (CHX) in DON-treated HT-22 cells. YTHDF2 knockdown significantly rectified the prolonged half-life of p21 protein in DON-treated cells (Fig. 6A), and such effect of YTHDF2 knockdown was abolished by the proteasome inhibitor MG132 (Fig. 6B, C). Further Co-IP analysis revealed significantly reduced ubiquitination level on p21 protein in DON-treated cells, which was significantly alleviated by YTHDF2 knockdown (Fig. 6D, E). These results suggest that YTHDF2 is presumably involved in m^6^A-mediated regulation of ubiquitination pathway.

TRIM21 E3 ligase promotes p21 ubiquitination in human neuroblastoma cells (Wang et al. 2021). To explore whether TRIM21 is involved in DON-induced decline in p21 ubiquitination in HT-22 cells, we determined TRIM21 mRNA and conducted its mRNA decay assay. Indeed, DON decreased TRIM21 mRNA (Fig. 6F) and accelerated its decay (Fig. 6G), and YTHDF2 knockdown significantly alleviated such effects of DON. We conducted an online search for potential m^6^A sites in the 3’UTR of TRIM21 mRNA. A specific m^6^A site (here refer as X1) was retrieved from a previous publication (Schwartz et al. 2014), and another m^6^A site (here refer as X2) was predicted using SRAMP (Fig. S6A and Supplementary Table 5). The levels of m^6^A modification on these two sites (X1, X2) were determined relative to the negative control site (N site). Only X2 site in p21 mRNA 3'UTR showed significantly increased level of m^6^A modification upon DON exposure which was prevented by METTL3 knockdown (Fig. 6H).

## Discussion

**Fig. 7:**
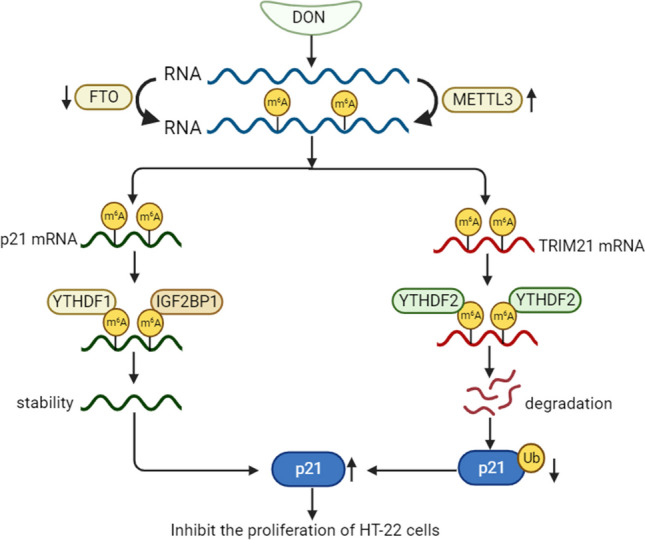
Proposed working model of the proposed mechanism in this study. Mechanism diagram of DON inhibits the proliferation of HT-22 cells. RNA m^6^A hypermethylation on the transcript of p21 enhances the mRNA stability in a YTHDF1- and IGF2BP1-dependent manner, which leads to the upregulation of p21. Additionally, RNA m^6^A hypermethylation on the transcript of the E3 ubiquitin ligase TRIM21 decreases the mRNA stability in a YTHDF2-dependent manner, which contributes to prevent p21 ubiquitin-mediated degradation**.** High expression of p21 contributes to inhibit cell proliferation

In this study, we present compelling evidence that DON induces G0/G1 cell cycle arrest in hippocampal HT-22 cells through upregulation of p21 via RNA m^6^A hypermethylation. We delineate the dual role of m^6^A in DON-induced upregulation of p21, operating at both post-transcriptional and post-translational levels, targeting different mRNAs, and involving distinct “reader” proteins. Specifically, YTHDF1 and IGF2BP1 contribute to the post-transcriptional upregulation of p21 via m^6^A-mediated increase in p21 mRNA stability, whereas YTHDF2 participates in the post-translational upregulation of p21 through m^6^A-dependent inhibition of TRIM21 mRNA stability and consequently impeding ubiquitin-mediated p21 protein degradation. Our findings underscore the intricate complexity of m^6^A-mediated gene regulation during DON-induced HT-22 cell cycle arrest (Fig. 7).

The cellular toxicity induced by mycotoxins includes the activation of apoptosis, the inhibition of cell proliferation, or the suppression of differentiation. For instance, ochratoxin A inhibits the proliferation and differentiation of hippocampal neural stem cells (Sava et al. 2007), and induces apoptosis in HT-22 cells (Yoon et al. 2009). In this study, both proliferation and apoptosis in HT-22 cells were evaluated, and no alterations were observed in the apoptosis rate. DON induced an inhibition of proliferation in HT-22 cells. The nature of toxicity depends on the type of cells and type of toxins, as well as the level and the duration of challenge. DON has been reported to inhibit cell proliferation through different mechanisms. DON inhibits proliferation by inhibiting the β-catenin/c-Myc axis in HEK 293 T and colon SW480 cells (Tang et al. 2019). In this study, DON-induced inhibition of HT-22 cell proliferation is accompanied by a significant upregulation of p21, which is consistent with a previous study in mouse thymic epithelial cells (Li et al. 2013). During cell proliferation, c-Myc functions as an accelerator, akin to a molecular throttle (Bretones et al. 2015), while p21 acts as a regulatory brake. p21 plays a key role at different checkpoints in the cell cycle, inducing arrest at G0/G1 (Napione et al. 2012) or G2/M (Tian et al. 2023) transition in response to DNA damage. G0/G1 arrest results from the checkpoint for S phase, while G2/M arrest attributes to the checkpoint for M phase. In this study, HT-22 cells were treated with DON (1 or 2 μM, i.e. 0.296 or 0.593 μg/mL) for 12 h. DON induces G0/G1 cell cycle arrest in HT-22 cells, which contradicts a previous study indicating that treatment of human colorectal carcinoma cells with DON (0, 0.5, and 1 μg/mL) for 48 h results in G2/M cell cycle arrest (Yang et al. 2008). HepG2 cells treated with DON (0, 0.5, 1, 2, and 4 μg/mL) for 6 h show G2/M cell cycle arrest, which is accompanied by the upregulation of p21 protein (Yuan et al. 2018). The difference in the phase of cell cycle arrest may be attribution to cell specificity, doses and duration of DON treatment.

As a key negative regulator of cell cycle progression (Karimian et al. 2016), p21 is subjected to complex regulations at different levels, among which RNA m^6^A modification (Ouyang et al. 2023) and protein ubiquitination (Zhang et al. 2019) are critical for regulating the stability of p21 mRNA and protein, respectively, at post-transcriptional and post-translational levels. For example, m^6^A hypermethylation on p21 mRNA increases p21 mRNA stability through IGF2BPs, leading to an inhibition of proliferation in PC9 cells (Tsuchiya et al. 2022). Also, knockdown of E3 ubiquitin ligase TRIM21 impairs ubiquitination-mediated p21 degradation, reducing the cell viability of human neuroblastoma cells (Wang 2021). In this study, m^6^A hypermethylation increases p21 mRNA stability via YTHDF1 and IGF2BP1, and simultaneously decreases TRIM21 mRNA stability via YTHDF2. The reduction of TRIM21 reduces the ubiquitination of p21, resulting in high p21 expression. High expression of p21 may play an accessory role in inhibiting cell proliferation. Collectively, we show for the first time that RNA m^6^A modification and protein ubiquitination are simultaneously involved in the regulation of p21.

Studies have shown that there is a crosstalk between RNA m^6^A modification and protein ubiquitination (Shi et al. 2023; Zhang et al. 2022b), but the relationship between RNA m^6^A modification and protein ubiquitination is not clear. In our study, we found that m^6^A was located upstream of ubiquitination. Then there were reports that FTO can be degraded by ubiquitination, thereby increasing m^6^A modification (Ruan et al. 2021). This may be related to cell types and the treatment of the model. In our model, METTL3 was upregulated and FTO was downregulated, enhanced the m^6^A modification of the TRIM21 3’UTR, and promoted mRNA degradation via YTHDF2 leading to low TRIM21 expression. Low expression of TRIM21 may play an accessory role in ubiquitination reduction of p21. A similar conclusion was obtained in a model of the proliferation in ESCC cells, where FTO reduced the m^6^A modification of LncRNA LINC00022, and inhibited LINC00022 decay via YTHDF2, contributing to LINC00022 directly binding to p21 protein and promoting its ubiquitination-mediated degradation (Cui et al. 2021). Although similar results were obtained, the enzymes involved were not the same, we have identified for the first time that m^6^A regulates p21 ubiquitination by regulating E3 ubiquitin ligase. This discovery sheds new light on the intricate molecular pathways governing p21 regulation, providing valuable insights into the nuanced mechanisms at play in cellular processes.

In our study, DON-induced cell cycle arrest via upregulation of p21 can be alleviated by inhibition of m^6^A hypermethylation. However, the intricacies of cellular metabolism are profound, and we cannot exclude the possibility that DON might influence cell proliferation through alternative pathways, which requires further comprehensive research. On the other hand, HT-22 cells are commonly used as a hippocampal neuronal cell model for mechanistic studies of neurotoxicity. Although in vitro cell culture models can not directly translate to the in vivo scenarios, our results provide a theoretical foundation for animal research. Future in vivo studies will thus extend these in vitro experiments and are required to uncover novel therapeutic avenues for the epigenetic therapy of neurodegenerative diseases. In conclusion, we provide evidence that the mechanism of m^6^A not only directly promotes p21 expression at the post-transcriptional level, but also promotes p21 expression at the post-translational level by regulating ubiquitination during DON inhibited proliferation of HT-22 cells. m^6^A can be used as the key targets for the regulation of p21. The insights derived from this research not only enhance our understanding of the intricate molecular mechanisms governing cell proliferation but also provide a novel avenue for advancing therapeutic strategies in the treatment of neurodegenerative diseases.

## Supplementary Information

Below is the link to the electronic supplementary material.

Supplementary file1 (DOCX 3064 KB)

## Data Availability

No datasets were generated or analysed during the current study.
