# Supplementary material for: Deoxynivalenol induces m6A-mediated upregulation of p21 and growth arrest of mouse hippocampal neuron cells in vitro
Source: Cell Biol Toxicol. 2024 Jun 4;40(1):41. doi: 10.1007/s10565-024-09872-7 (PMC11150311; doi:10.1007/s10565-024-09872-7)
Supplement: Supplementary file 1 — Supplementary file1 (DOCX 3064 KB) [file 10565_2024_9872_MOESM1_ESM.docx]

**Supplementary information**

**Deoxynivalenol induces m^6^A-mediated upregulation of p21 and growth arrest of mouse hippocampal neuron cells in vitro**

Peirong Xu^1,2^, Yulan Zhao^1,2^, Yue Feng^1,2^, Mindie Zhao^1,2^, Ruqian Zhao^1,2,*^

^1^MOE Joint International Research Laboratory of Animal Health & Food Safety, Nanjing Agricultural University, Nanjing, Jiangsu, P. R. China.

^2^Key Laboratory of Animal Physiology & Biochemistry, College of Veterinary Medicine, Nanjing Agricultural University, Nanjing, Jiangsu, P. R. China.

*Correspondence: Professor Ruqian Zhao, Key Laboratory of Animal Physiology & Biochemistry, Nanjing Agricultural University, Nanjing, Jiangsu, P. R. China. E-mail: zhaoruqian@njau.edu.cn.

**Supplementary figures and legends**

**
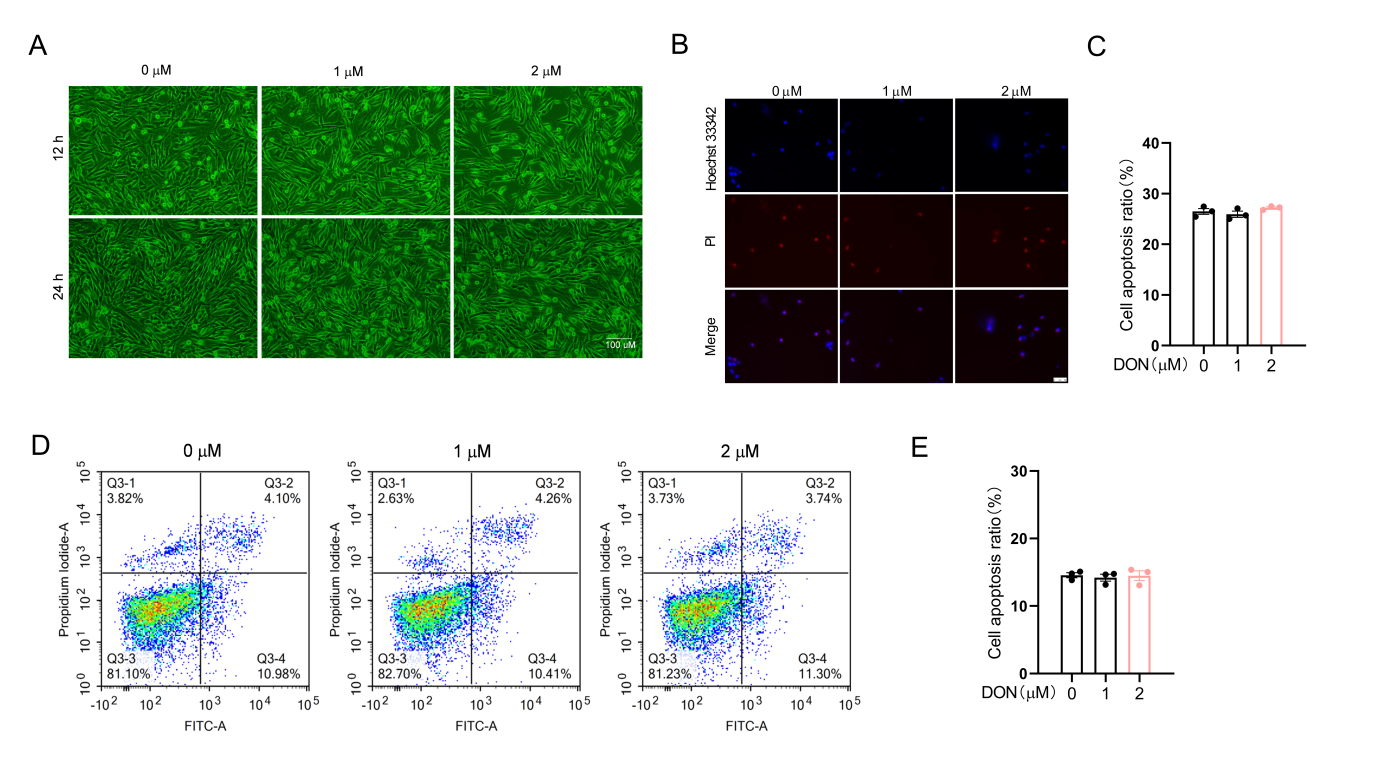
**

**Supplementary Fig. 1. Effect of apoptosis in DON-treated HT-22 cells.** HT-22 cells were treated with DON (1 or 2 μM) for 12 h. A Cell morphological changes. B-E Flow cytometry and fluorescence microscopy assays for cell apoptosis ratio (n = 3). The differences between groups were analyzed using one-way ANOVA with Bonferroni’s correction. Values are means ± SD, **P* < 0.05, ***P* < 0.01.

**
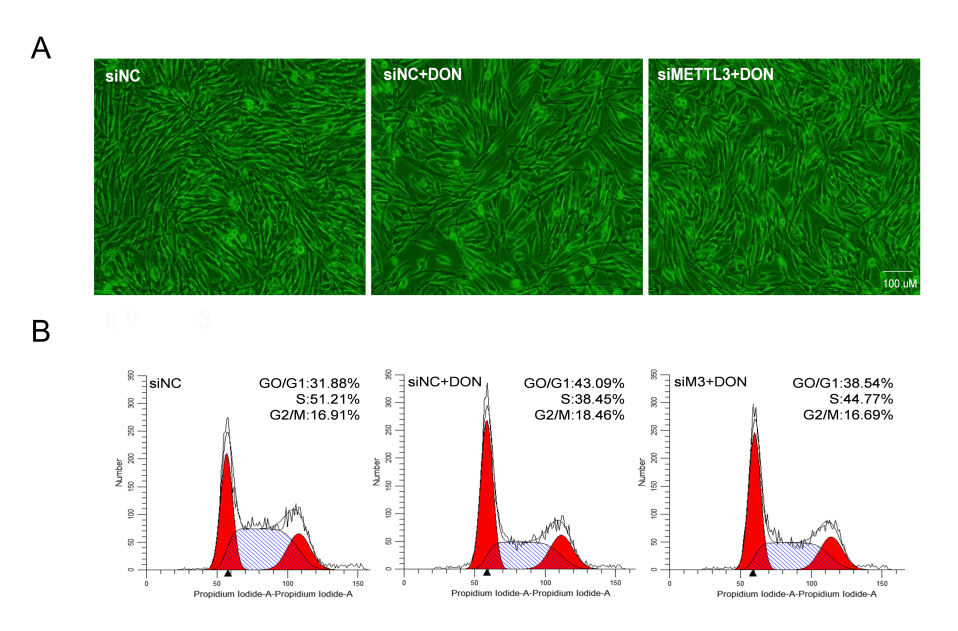
**

**Supplementary Fig. 2. Effect of METTL3 siRNA on cell morphological and cell cycle in DON-treated HT-22 cells.** HT-22 cells were treated with DON (2 μM) for 12 h. **A** Effect of METTL3 siRNA on cell morphological changes (*n* = 3). **B** Statistical analysis of effect of METTL3 siRNA on cell cycle distribution (*n* = 3).

**
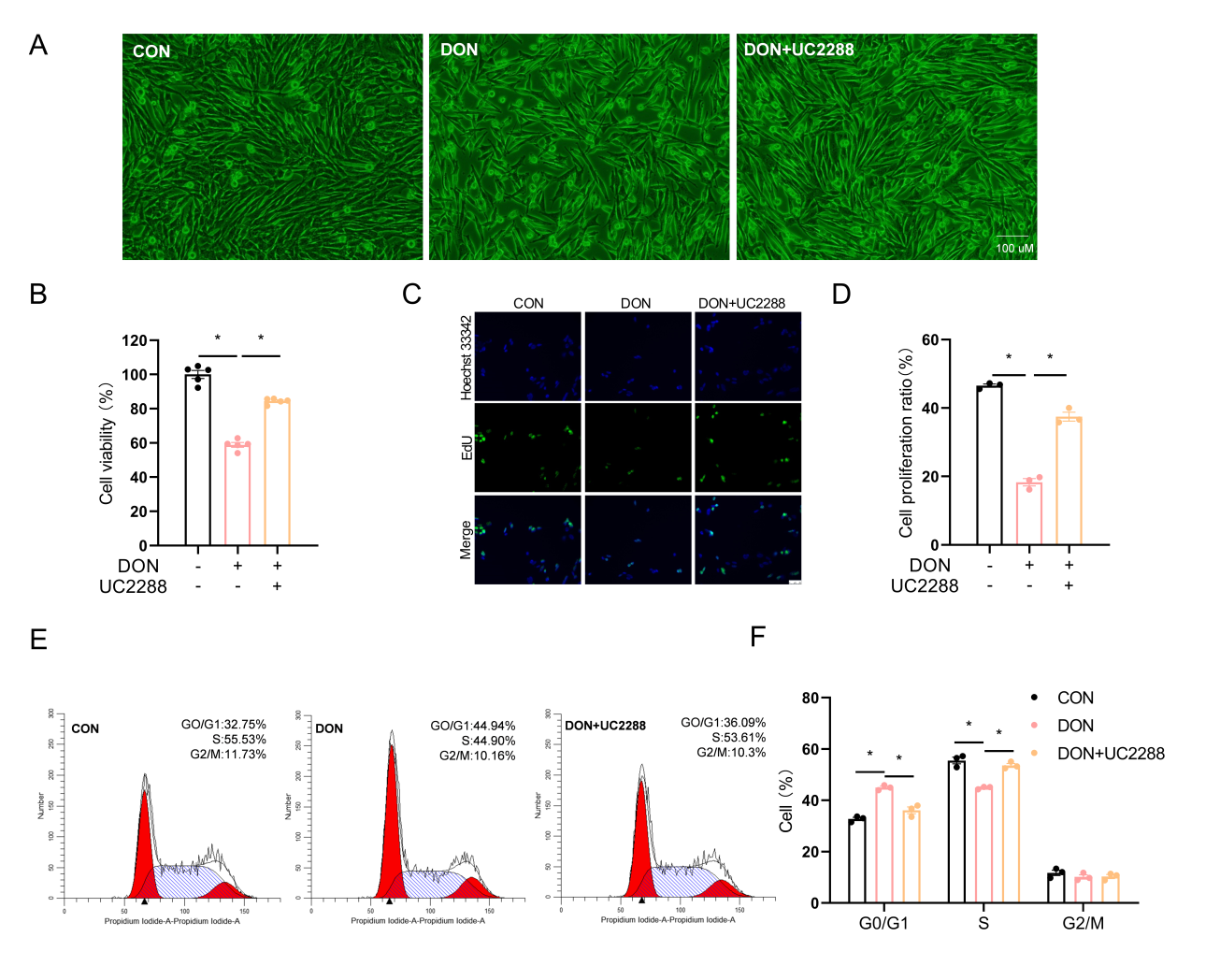
**

**Supplementary Fig. 3. Effect of UC2288 on cell morphological, cell viability, cell proliferation and cell cycle in DON-treated HT-22 cells.** HT-22 cells were treated with DON (2 μM) for 12 h. **A** Effect of UC2288 on cell morphological changes (*n* = 3). **B** Effect of UC2288 on cell viability (*n* = 3). **C**, **D** Effect of UC2288 on cell proliferation ratio (*n* = 3). **E**, **F** Effect of UC2288 on cell cycle distribution (*n* = 3). The differences between groups were analyzed using one-way ANOVA with Bonferroni’s correction. Values are means ± SD, **P* < 0.05.

**
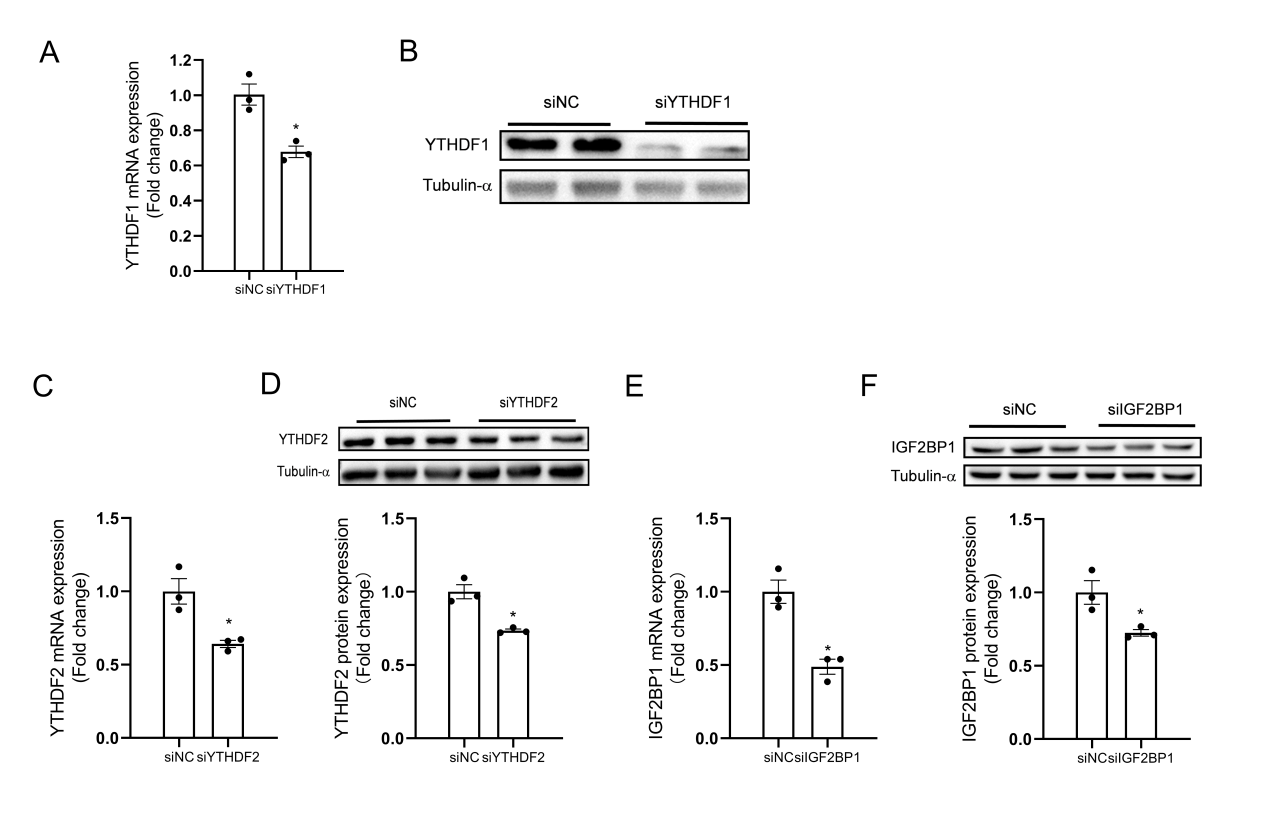
**

**Supplementary Fig. 4. Validation of siRNA transfection efficiency in HT-22 cells. A** Inhibition of YTHDF1 mRNA expression by YTHDF1 siRNA transfection (*n* = 3). **B** Inhibition of YTHDF1 protein expression by YTHDF1 siRNA transfection (*n* = 2). **C**, **D** Inhibition of YTHDF2 mRNA and protein expression by YTHDF2 siRNA transfection (*n* = 3). **E**, **F** Inhibition of IGF2BP1 mRNA and protein expression by IGF2BP1 siRNA transfection (*n* = 3). The differences between groups were analyzed using student’s t test. Values are means ± SD, **P* < 0.05.

**
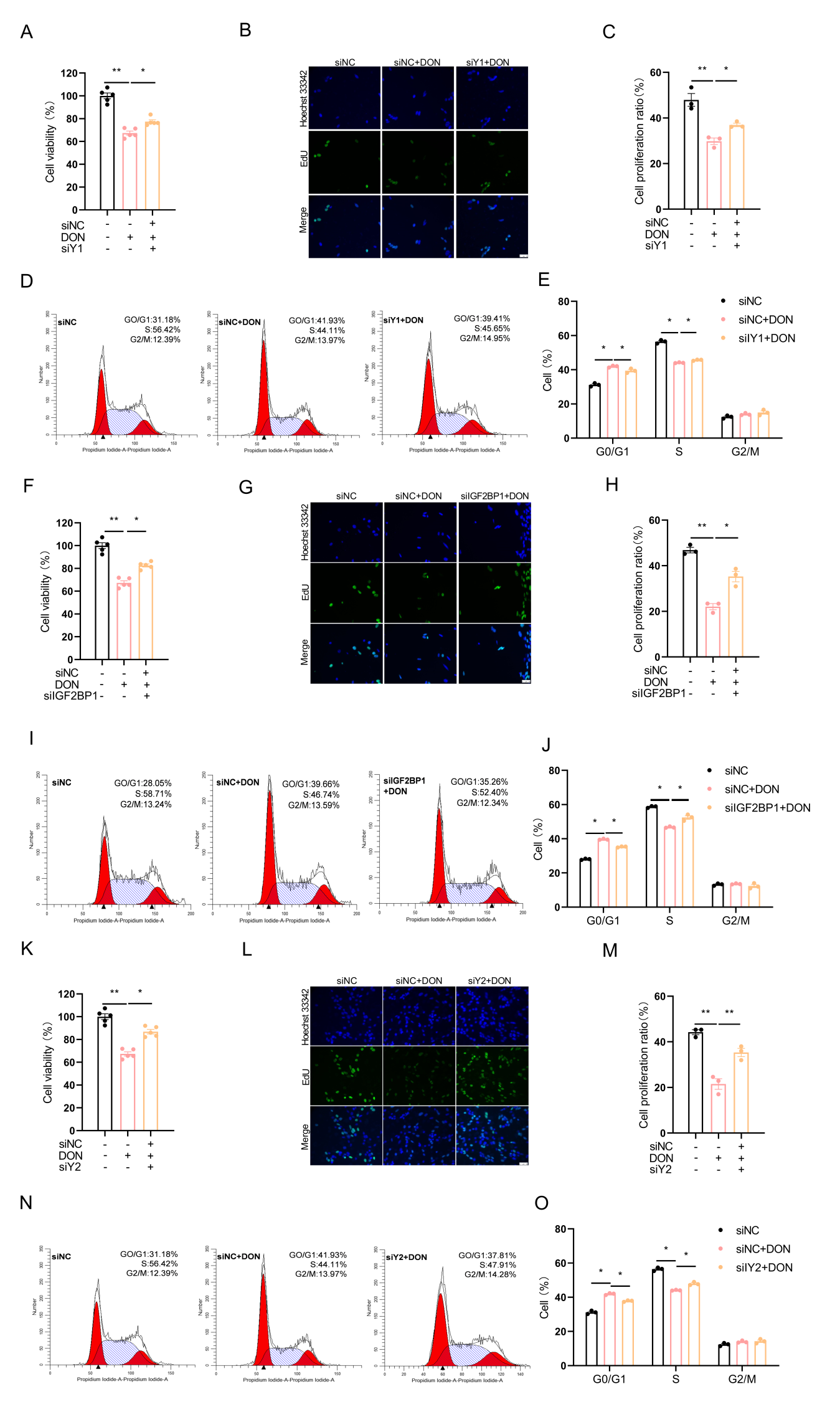
**

**Supplementary Fig. 5. Effects of individual knockdown of YTHDF1, YTHDF2, or IGF2BP1 on cell viability, cell proliferation and cell cycle in DON-treated HT-22 cells.** HT-22 cells were treated with DON (2 μM) for 12 h. **A** Effect of YTHDF1 siRNA on cell viability (*n* = 3). **B**, **C** Effect of YTHDF1 siRNA on cell proliferation ratio (*n* = 3). **D**, **E** Effect of YTHDF1 siRNA on cell cycle distribution (*n* = 3). **F** Effect of IGF2BP1 siRNA on cell viability (*n* = 3). **G**, **H** Effect of IGF2BP1 siRNA on cell proliferation ratio (*n* = 3). **I**, **J** Effect of IGF2BP1 siRNA on cell cycle distribution (*n* = 3). **K** Effect of YTHDF2 siRNA on cell viability (*n* = 3). **L**, **M** Effect of YTHDF2 siRNA on cell proliferation ratio (*n* = 3). **N**, **O** Effect of YTHDF2 siRNA on cell cycle distribution (*n* = 3). The differences between groups were analyzed using one-way ANOVA with Bonferroni’s correction. Values are means ± SD, **P* < 0.05, ***P* < 0.01.

**
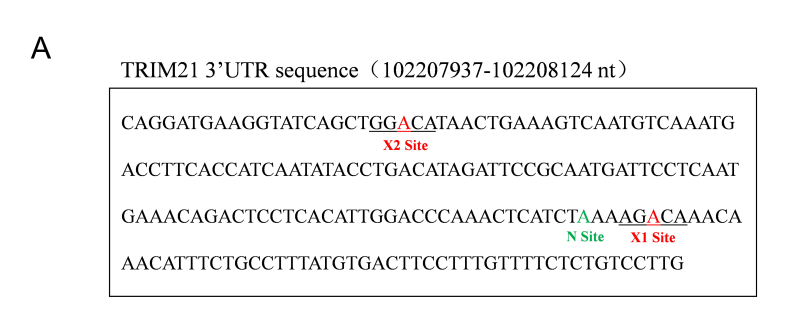
**

**Supplementary Fig. 6. Potential m^6^A site in TRIM21 3’UTR. A** Online search for potential m^6^A sites in 3’UTR (102207937-102208124 nt) of TRIM21 mRNA. A specific m^6^A site (X1) was retrieved from published databases (https://rmvar.renlab.org/), and another m^6^A site (X2) was predicted using SRAMP (http://www.cuilab.cn/sramp). The non-modification A was named N site.


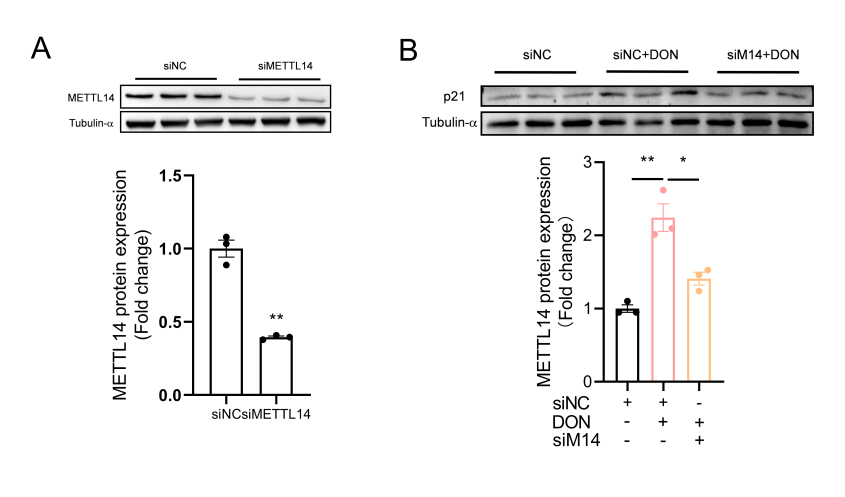


**Supplementary Fig. 7. Effect of METTL14 siRNA on METTL14 and p21 protein expression.** HT-22 cells were treated with DON (2 μM) for 12 h. **A** Inhibition of METTL14 protein expression by METTL14 siRNA transfection (*n* = 3). The differences between groups were analyzed using student’s t test. **B** Effect of METTL14 siRNA on p21 protein expression (*n* = 3). The differences between groups were analyzed using one-way ANOVA with Bonferroni’s correction. Values are means ± SD, **P* < 0.05, ***P* < 0.01.

**Supplementary table 1.** Nucleotide sequences of primers for Real-time PCR

| Target genes | Primer sequences (5’ to 3’) | Used for |
| --- | --- | --- |
| p53 | F: CACCTCACTGCATGGACGAT | Real-time PCR |
|  | R: GTGGAAGCCCATGTTGCCCT |  |
| CDKN1A | F: CCCGAGAACGGTGGAACTTT | Real-time PCR |
|  | R: AGAGTGCAAGACAGCGACAA |  |
| METTL3 | F: CAGTGCTACAGGATGACGGCTT | Real-time PCR |
|  | R: CCGTCCTAATGATGCGCTGCAG |  |
| METTL14 | F: AGAGTGCGGATAGCATTGGTGC | Real-time PCR |
|  | R: CTCCTTCATCCAGACACTTCCG |  |
| FTO | F: GCCTCGGTTTAGTTCCACTCAC | Real-time PCR |
|  | R: GTCGCCATCGTCTGAGTCATTG |  |
| YTHDF1 | F: GCATCAGAAGGATGCAGTTCATG | Real-time PCR |
|  | R: GATGGTGGATAGTAACTGGACAG |  |
| YTHDF2 | F: GGTTCTGTGCATCAAAAGGATGG | Real-time PCR |
|  | R: CCAAAGAATAGGAAAAGCCAATGG |  |
| YTHDF3 | F: GGTTCGATTCATCAAAAAGATGCTG | Real-time PCR |
|  | R: GATCTGACATTGGTGGATAGCTG |  |
| IGF2BP1 | F: CCCGCAGACTTGGAGAAAGT | Real-time PCR |
|  | R: CACTTCCCATCGGAGCTGAG |  |
| TRIM21 | F: GTTTTTGCTGGACCTCTGCG | Real-time PCR |
|  | R: AGCTTTAGAGGCGCTGCATT |  |
| PPIA | F: CATACAGGTCCTGGCATCTTGTC | Real-time PCR |
|  | R: AGACCACATGCTTGCCATCCAG |  |

| Antibodies | Source | Catalogue NO. | Dilution |
| --- | --- | --- | --- |
| p53 | Bioworld | BS6437 | 1:500 |
| CDKN1A | Proteintech | 10355-1-AP | 1:500 |
| METTL3 | Abcam | AB98009 | 1:1000 |
| METTL14 | Abcam | AB98116 | 1:1000 |
| FTO | Abcam | AB77547 | 1:1000 |
| YTHDF1 | Proteintech | 17479-1-AP | 1:2000 |
| YTHDF2 | Proteintech | 24744-1-AP | 1:2000 |
| YTHDF3 | Proteintech | 25537-1-AP | 1:2000 |
| IGF2BP1 | Proteintech | 22803-1-AP | 1:2000 |
| Tubulin-α | Bioworld | BS1699 | 1:10000 |

**Supplementary table 2.** The list of antibodies**Supplementary table 3.** Nucleotide sequences of SELECT method

| Target | Sequences (5’ to 3’) |
| --- | --- |
| CDKN1A  X1 site | Up Probe: tagccagtaccgtagtgcgtgTGCGGGCTAAGGGTAGACAG |
|  | Down Probe: CCAGACCAGGATGTTACAGAcagaggctgagtcgctgcat |
| CDKN1A  X2 site | Up Probe: tagccagtaccgtagtgcgtgGCATATACATTCCCTTCCAG |
|  | Down Probe: CCACTGAGCTGTGGGGCAAGcagaggctgagtcgctgcat |
| CDKN1A  X3 site | Up Probe: tagccagtaccgtagtgcgtgGTCGGGATATTACGGTTGAG |
|  | Down Probe: CCTAACTGCCATCCCTGTTCcagaggctgagtcgctgcat |
| CDKN1A  X4 site | Up Probe: tagccagtaccgtagtgcgtgGTGACAAGGAGACCCCAAAG |
|  | Down Probe: CCTACTCATTTTTCCAAAGTGcagaggctgagtcgctgcat |
| CDKN1A  N site | Up Probe: tagccagtaccgtagtgcgtgGCTAAGGGTAGACAGTCCAGA |
|  | Down Probe: CAGGATGTTACAGAAACAGGcagaggctgagtcgctgcat |
| TRIM21  X1 site | Up Probe: tagccagtaccgtagtgcgtgAGGCAGAAATGTTTGTTTGT |
|  | Down Probe: CTTTTAGATGAGTTTGGGTCCcagaggctgagtcgctgcat |
| TRIM21  X2 site | Up Probe: tagccagtaccgtagtgcgtgTTGACATTGACTTTCAGTTATG |
|  | Down Probe: CCAGCTGATACCTTCATCCTcagaggctgagtcgctgcat |
| TRIM21  N site | Up Probe: tagccagtaccgtagtgcgtgGCAGAAATGTTTGTTTGTCTTT |
|  | Down Probe: AGATGAGTTTGGGTCCAATGcagaggctgagtcgctgcat |
| qPCR | Forward Prime: ATGCAGCGACTCAGCCTCTG |
|  | Reverse Prime: TAGCCAGTACCGTAGTGCGTG |

**Supplementary table 4.** m^6^A site was predicted in SRAMP for p21 3’UTR

| Site | Position (nt) | Sequence context | Score | Decision |
| --- | --- | --- | --- | --- |
| X1 | 29319973 | GGACT | 0.599 | m^6^A site (moderate confidence) |
| X2 | 29319394 | GGACT | 0.656 | m^6^A site (high confidence) |
| X3 | 29319347 | GGACT | 0.688 | m^6^A site (very high confidence) |
| X4 | 29319264 | GGACT | 0.685 | m^6^A site (very high confidence) |

**Supplementary table 5.** m^6^A site was predicted in SRAMP for TRIM21 3’UTR

| Site | Position | Sequence context | Score | Decision |
| --- | --- | --- | --- | --- |
| X2 | 102208162 | GGACA | 0.608 | m^6^A site (high confidence) |
